# The Current Landscape of Metastatic Breast Cancer: A Pathology Guide on Emerging Biomarkers

**DOI:** 10.3390/cancers18101544

**Published:** 2026-05-10

**Authors:** Joana Ferreira, André Albergaria, Fernando Schmitt

**Affiliations:** RISE-Health, Department of Pathology, Faculty of Medicine, University of Porto, 4200-319 Porto, Portugal; up202208563@up.pt (J.F.); aalbergaria@med.up.pt (A.A.)

**Keywords:** metastatic breast cancer, biomarkers, precision oncology, HER2-low, liquid biopsy, PIK3CA, PD-L1, pathologist role, artificial intelligence

## Abstract

**Background/Objectives**: Metastatic breast cancer (MBC) remains a daunting clinical challenge, accounting for approximately 90% of all breast cancer-related deaths. The management of MBC has shifted from traditional chemotherapy to a sophisticated, biomarker-driven model of precision oncology. This evolution is predicated on the ability of biomarkers to provide prognostic and predictive information. The objective of this review is to provide a comprehensive synthesis of the current landscape of biomarker testing in MBC, detailing which biomarkers to test and the clinical rationale for doing so. **Methods**: This is a comprehensive review based on current international clinical practice guidelines, peer-reviewed literature, and evidence regarding clinically actionable and emerging biomarkers in metastatic breast cancer. **Results**: Key biomarkers currently in routine use include the established estrogen receptor (ER), progesterone receptor (PR), and HER2, alongside newer, clinically actionable alterations such as mutations in PIK3CA and ESR1, germline/somatic BRCA1/2, and PD-L1 expression. Furthermore, liquid biopsy, particularly the analysis of circulating tumor DNA (ctDNA), is rapidly gaining prominence as a non-invasive tool for real-time disease monitoring and resistance detection, highlighting the critical need for re-testing at metastasis due to tumor heterogeneity. **Conclusions**: The future of personalized oncology in MBC will be defined by the seamless integration of dynamic biomarkers and cutting-edge technologies. The integration of AI and spatial transcriptomics will move the field of pathology beyond a static diagnosis to a more dynamic and predictive model, reinforcing the pathologist’s role as the “molecular gatekeeper” for adaptive and personalized cancer care.

## Simple Summary

Metastatic breast cancer (MBC) is the leading cause of death in breast cancer patients. Successfully managing this disease requires biomarkers to identify the tumor’s vulnerabilities; yet a major challenge is the molecular discordance found between primary tumors and metastatic lesions. This review serves as a guide for pathologists and oncologists, detailing which essential biomarkers to test—such as ER/PR, HER2, and key genetic mutations like PIK3CA and ESR1—while emphasizing the critical need for re-biopsy or liquid biopsy in the metastatic setting to capture the tumor’s clonal evolution. We also explore advanced non-invasive methods for real-time disease monitoring and discuss the future role of Artificial Intelligence (AI) in integrating complex data. The ultimate goal is to move from a standard treatment model to a dynamic, precision approach, ensuring each patient receives the most effective drug based on the current, evolving biology of their tumor lesions.

## 1. Introduction

### 1.1. Metastatic Breast Cancer Epidemiology and Prognosis

Metastatic breast cancer (MBC) is an advanced stage of the disease and the primary cause of breast cancer-related mortality. Globally, the burden is rising; in 2022, there were 2.3 million new breast cancer cases and 666,103 deaths reported by GLOBOCAN. While MBC remains incurable, modern therapies have transformed it into a complex chronic condition with prolonged survival [1]. MBC arises through two distinct pathways: as a *de novo* diagnosis in approximately 5–10% of new cases [2] or, more commonly, as a distant recurrence in 20–30% of patients initially diagnosed with early-stage disease [3].

While MBC is considered incurable, the prognosis has improved, with the overall 5-year relative survival rate now standing at approximately 32% [4]. However, this single figure masks a profound clinical and biological heterogeneity, which is the central clinical challenge in managing the disease. The most critical prognostic determinant is the molecular subtype, defined by the status of hormone receptors (HR) and human epidermal growth factor receptor 2 (HER2). Survival outcomes vary dramatically across these subtypes, underscoring the pathologist’s essential role in biomarker assessment. As detailed in Table 1, recent data from SEER (Surveillance, Epidemiology, and End Results) illustrate how dramatically 5-year relative survival rates for distant-stage disease vary by subtype [4].

These survival disparities are largely influenced by the therapeutic arsenal available for each molecular subtype. The superior outcomes observed in HR+/HER2+ metastatic breast cancer (46.7% 5-year survival) are primarily attributed to the availability of both hormone therapies and HER2-targeted treatments, which can be highly effective when used in combination. This contrasts with HR+/HER2− tumors (36.5% 5-year survival), which, although hormone-driven, lack the additional clinical benefit provided by HER2-directed targeted therapy options.

Prognosis is further stratified by the site and burden of metastatic disease. Patients with bone-only metastases, for instance, have a more favorable clinical outcome (39.8% 5-year survival) compared to those with visceral or brain metastases (1.51% 5-year survival) [5]. Contributing to this complexity are persistent and significant racial disparities. Black women face a 41% higher mortality rate than white women. Structural racism drives chronic stress, resulting in inflammation and accelerated cellular aging. This biological process is associated with more frequent diagnoses of aggressive tumor biologies, such as the triple-negative subtype [6].

This multifaceted landscape highlights the imperative for precise biomarker analysis to guide personalized therapeutic strategies and improve patient outcomes.

### 1.2. Evolution of Biomarker-Guided Therapy in Breast Cancer

The historical approach to breast cancer treatment was rooted in an empiric, non-specific modality. This was exemplified by the use of combination chemotherapy regimens like CMF (cyclophosphamide, methotrexate, and fluorouracil) in the 1970s, which were applied irrespective of the tumor’s underlying biology [7]. This era of “one-size-fits-all” medicine has given way to a new paradigm—precision oncology—a transformation driven by the discovery and clinical validation of actionable biomarkers [7]. The conceptual origins of targeted therapy trace back to 1896, when George Beatson demonstrated that oophorectomy could lead to tumor regression, though the mechanism was unknown [8,9]. The molecular era truly began with the discovery of the estrogen receptor (ER) in 1958, which culminated in the FDA’s approval of the first selective estrogen receptor modulator (SERM), tamoxifen, in 1978 [10]. The second pivotal moment came with the identification of the *ERBB2* gene (HER2) in the mid-1980s and the subsequent FDA approval of the monoclonal antibody trastuzumab in 1998 [11,12]. The validation of ER, progesterone receptor (PR), and HER2 as predictive biomarkers allowed for the classification of breast cancer into distinct molecular subtypes and enabled the development of the first generation of targeted therapies.

The introduction of these targeted agents was a pivotal event that fundamentally reshaped the therapeutic landscape. It became clear that breast cancer was not a singular disease but a vast group of molecularly distinct entities, each requiring a different therapeutic approach [7]. This understanding created a co-dependent ecosystem between drug development and biomarker discovery. Recognizing this shift, the FDA developed a framework to qualify biomarkers and has since approved dozens of oncologic drugs based on the presence or absence of a specific biomarker, bolstering the link between precise molecular diagnosis and tailored treatment selection [13]. Specific examples of this biomarker–drug co-dependency include the approval of inavolisib alongside the FoundationOne Liquid CDx for PIK3CA mutations [14], and the use of elacestrant and imlunestrant linked to the Guardant360 CDx for identifying ESR1 mutations [15]. In the HER2 setting, the expansion of trastuzumab deruxtecan into the HER2-low population was supported by the co-approval of the PATHWAY anti-HER2/neu (4B5) diagnostic tool [16]. This evolution is grounded on the ability of biomarkers to provide both prognostic information (predicting outcome independent of treatment) and predictive information (predicting response to a specific therapy) [17]. This biomarker-driven evolution represents the core of precision oncology: it is not just about treating the cancer, but about treating the right patient with the right therapy. This represents a profound conceptual shift from traditional oncology, where treatment was primarily based on anatomical staging, and moving toward a future where therapeutic decisions are guided by a deep understanding of the tumor’s specific genetic and molecular vulnerabilities.

In recent years, the arsenal of actionable biomarkers in MBC has expanded significantly beyond ER, PR, and HER2. Key additions that are now standard of care include:

- Germline *BRCA1/2* Mutations: Pathogenic mutations in these genes confer sensitivity to PARP inhibitors, leading to the approval of drugs like olaparib for patients with HER2-negative MBC [18,19].
- Somatic Mutations in HR-positive, HER2-negative MBC:

○ *PIK3CA* Mutations: These are targeted by PI3Kα inhibitors, such as alpelisib and the recently approved inavolisib (October 2024) [14,20].
○ *ESR1* Mutations: These acquired mutations are a mechanism of resistance to endocrine therapy and are targeted by oral SERDs, such as elacestrant and imlunestrant [15,21].

- *AKT1*/*PTEN* Alterations: Alterations in the PI3K/AKT pathway are targeted by AKT inhibitors, such as capivasertib, approved in late 2023 [22].
- PD-L1 Expression: In triple-negative breast cancer (TNBC), the expression of PD-L1 (measured by a Combined Positive Score ≥10) is a predictive biomarker for response to the immune checkpoint inhibitor pembrolizumab in combination with chemotherapy [23].

Furthermore, the field is advancing with emerging biomarkers and new classifications. The refinement of the HER2 category to include “HER2-low” has created a new therapeutically relevant subgroup, enabling the use of antibody–drug conjugates like trastuzumab deruxtecan for a much larger patient population [24]. Concurrently, the use of circulating tumor DNA (ctDNA) via liquid biopsy is emerging as a powerful, non-invasive tool for monitoring treatment response, detecting minimal residual disease, and identifying the emergence of resistance mutations in real time, promising an even more dynamic and personalized future for MBC management [13,17].

## 2. The Molecular Landscape of Metastatic Breast Cancer

### 2.1. Heterogeneity of MBC: Molecular Subtypes and Clonal Evolution

A hallmark of cancer, particularly for MBC, is its intrinsic heterogeneity [25,26]. This heterogeneity exists on multiple levels: between different patients (inter-patient heterogeneity), between a primary tumor and its metastases or between different metastatic lesions within the same patient (inter-lesion heterogeneity), and even within a single tumor mass (intra-tumor heterogeneity) [27]. This complexity is further heightened by the dynamic process of clonal evolution, a key mechanism by which tumors adapt to therapeutic pressure, developing multiple mechanisms of resistance [28]. Clonal evolution involves the accumulation of new genetic and epigenetic alterations in a subset of cancer cells, leading to the emergence of new subclones that can outcompete and replace the original tumor population [25].

This heterogeneity has profound clinical implications. The discordance of key biomarkers between a primary tumor and its metastatic recurrence is a well-documented phenomenon. For this reason, major clinical guidelines, such as those from ESMO, recommend that patients with newly diagnosed or recurrent MBC should have a metastatic lesion biopsied, if technically feasible, to re-assess estrogen receptor (ER), progesterone receptor (PR), and HER2 status [29]. Treatment decisions should be guided by the biomarker profile of the metastatic disease, as it may have evolved from the primary tumor [29].

Studies analyzing clonal evolution patterns, often via circulating tumor DNA (ctDNA), have elucidated two primary models: linear and branched evolution [28]. In a linear model, a single, dominant clone acquires sequential mutations that drive disease progression, often leading to rapid and aggressive resistance [30,31]. In contrast, the branched model involves a primary clone that gives rise to multiple competing subclones, each with a different set of mutations [30]. The complex evolution of these tumor subclones can be visualized using an analogy to a “family tree” or phylogenetic tree, where the trunk represents the earliest, shared mutations and the branches represent the diverse, evolving subclones that may contribute to resistance and metastasis [32].

Recent evidence suggests the branched evolution pattern is associated with a slower rate of disease progression and a better prognosis than the linear model [28]. While a large 2025 study found that most MBCs overall exhibited a branched evolution pattern [28], these dynamics are known to differ by subtype. For example, triple-negative breast cancers (TNBCs) have been shown to have increased mutation rates compared to HR-positive tumors, which contributes to their distinct evolutionary trajectories and aggressive clinical outcomes [33]. These dynamics suggest that the pattern of tumor evolution itself can serve as a powerful, real-time prognostic biomarker, offering a more profound level of insight than traditional static markers alone and potentially guiding the intensity of therapeutic interventions [28].

The use of ctDNA via liquid biopsy is emerging as a critical tool for non-invasively monitoring these clonal dynamics, detecting the emergence of resistance mutations (such as in *ESR1*), and potentially guiding the selection of therapeutic interventions in real time [34]. This approach is being actively investigated in large-scale studies like the PADA-1 trial, which aims to adapt treatment plans based on real-time biomarker data as a patient’s tumor changes [35].

From a clinical perspective, not all forms of heterogeneity carry the same therapeutic weight. Truncal alterations, such as PIK3CA mutations, are typically early events and remain stable across disease progression, whereas branch-specific or acquired alterations, such as ESR1 mutations, arise under therapeutic pressure and directly mediate treatment resistance. This distinction underscores why static biomarker assessment at diagnosis is insufficient in metastatic disease and highlights the need for longitudinal profiling strategies capable of capturing dynamic clonal shifts [28].

### 2.2. Differences Between Primary and Metastatic Disease

The dynamic nature of clonal evolution creates a significant challenge for clinical practice: the molecular profile of a metastatic lesion may be substantially different from that of the primary tumor from which it originated [3]. A single tissue biopsy of the primary tumor, performed at initial diagnosis, may not provide an accurate or comprehensive representation of the disease characteristics at the time of metastasis [36].

Numerous studies have demonstrated this, reporting significant discordance rates for key biomarkers. For example, recent meta-analyses report pooled discordance rates of approximately 19–20% for ER, 33–34% for PR, and 8–15% for HER2 [37,38]. This discordance is not a random occurrence but often a direct consequence of the selective pressure exerted by adjuvant therapies, which favor the survival of subclones with new resistance-driving mutations [38]. This is a crucial point that has led major clinical guidelines, such as those from ESMO, to strongly recommend re-biopsy of a metastatic lesion whenever feasible to ensure treatment decisions are based on the current biology of the tumor [29].

While many biomarkers evolve, some truncal mutations, like those in PIK3CA, are considered early events in tumorigenesis and show high concordance (approximately 90%) between primary and metastatic sites [39]. In contrast, other biomarkers are defined by their temporal instability. Activating mutations in ESR1, for example, are rare in primary tumors but are detected in up to 40% of ER-positive metastatic cases after endocrine therapy, conferring resistance [36,40]. Similarly, newer classifications such as HER2-low and PD-L1 expression are also highly dynamic, with discordance rates reported as high as ~50% and ~28–41%, respectively, directly impacting eligibility for novel antibody–drug conjugates and immunotherapies [41,42].

## 3. Clinically Actionable Biomarkers in Routine Practice

### 3.1. Pathological Classification of Molecular Subtypes

The molecular classification of breast cancer, traditionally based on the immunohistochemical (IHC) expression of hormone receptors (ER, PR) and HER2, remains the cornerstone of clinical decision-making and dictates systemic therapy [29]. These subtypes are defined by clear pathological cutoffs:

- Hormone Receptor-Positive (HR+): Defined as tumors expressing ER and/or PR in ≥1% of tumor cells by IHC [43]. These biomarkers are both prognostic (conferring a more favorable prognosis) and predictive of response to endocrine therapy [13,44].
- HER2-Positive (HER2+): Defined by an IHC score of 3+ (strong, complete membrane staining) or an IHC score of 2+ (equivocal) with ERBB2 gene amplification confirmed by in situ hybridization (ISH) [43]. This status is predictive of response to HER2-directed therapies, such as trastuzumab [13].
- Triple-Negative Breast Cancer (TNBC): Tumors lacking expression of ER and PR (defined as <1% staining) and HER2 (IHC 0, 1+, or 2+/ISH-negative) [38].
- A recent paradigm shift, cemented by the 2023 FDA approval of the antibody–drug conjugate (ADC) trastuzumab deruxtecan (T-Dxd) for the HER2 group, is the re-evaluation of HER2-negative disease [24]. This has established the new, therapeutically critical category of HER2-low [24]. These are tumors defined by an IHC score of 1+ or an IHC score of 2+ with a negative ISH result [41].
- The HER2-low category is estimated to include approximately 50–60% of all breast cancers, the majority of which were previously classified as HR+/HER2− [29,45]. It is crucial to note that HER2-low is not a distinct biological subtype but a therapeutic classification that makes a large patient population eligible for ADC therapy. This reclassification has placed new demands on pathologists to meticulously distinguish between HER2 IHC 0 (now termed “HER2-zero”) and HER2 IHC 1+ scores, a distinction that was previously clinically irrelevant but is now a critical therapeutic decision point [41]. Furthermore, this status is highly dynamic, with some studies reporting discordance rates of nearly 50% between primary and metastatic sites, further emphasizing the need to re-biopsy metastatic lesions [41,45].

### 3.2. Summary of Actionable Targets

The following section details the key biomarkers that have become standard of care in guiding therapeutic decisions for MBC. These are summarized in Table 2 and discussed individually below.

### 3.3. Hormone Receptors (ER, PR)

The status of estrogen (ER) and progesterone (PR) receptors, pathologically defined by a cutoff of ≥1% positive staining cells, is the foundational biomarker in breast cancer [62]. These markers predict response to endocrine therapies, which are the cornerstone of treatment for HR-positive MBC, typically combined with CDK4/6 inhibitors [29].

As previously discussed, biomarker status can change over time. This instability is why major guidelines mandate re-biopsy of metastatic lesions where feasible [29,63]. Data from recent meta-analyses show pooled discordance rates between the primary tumor and metastatic sites of approximately 19–20% for ER and 33–34% for PR, making PR the most unstable of the classic receptors [38,64]. This loss of positivity is more common than gain and is prognostically significant, as it is associated with worse overall survival [65].

### 3.4. HER2 (ERBB2)

The HER2 protein, encoded by the *ERBB2* gene, is a growth-promoting receptor [12]. Pathologic determination of HER2 status is a critical predictive biomarker.

The HER2-Positive subtype is defined by HER2 overexpression (IHC 3+) or gene amplification (IHC 2+/ISH+) [66]. It was historically a highly aggressive subtype, but the development of HER2-directed therapies, starting with the monoclonal antibody trastuzumab, revolutionized its treatment [11]. The long-standing first-line standard of care for metastatic HER2-positive disease was established by the CLEOPATRA trial, which combined dual HER2 blockade (trastuzumab and pertuzumab) with a taxane [67].

Therapeutic strategies targeting HER2 are still evolving with the recent DESTINY-Breast09 trial, presented at ASCO 2025, demonstrating that trastuzumab deruxtecan (T-DXd) plus pertuzumab significantly improved PFS over the CLEOPATRA regimen, establishing a new, chemotherapy-free benchmark for the HER2-positive breast cancer management [68]. While trastuzumab deruxtecan (T-DXd) has transformed the treatment of HER2-positive breast cancer, this shift is not without limitations. Although T-DXd reduces the systemic burden compared to traditional taxanes, it introduces specific safety concerns, most notably interstitial lung disease (ILD)/pneumonitis. In the first-line treatment setting, adjudicated drug-related ILD/pneumonitis occurs in approximately 12.1% of patients [69]. Long-term efficacy is hindered by resistance centered on target engagement loss. Approximately 49% of cases show significant HER2 downregulation at progression, with over half exhibiting complete loss (IHC 0). Furthermore, ERBB2 mutations (e.g., V597M, P593R) at the binding interface mechanically impair drug attachment. These alterations reduce drug internalization, preventing the payload from reaching its target and enabling tumor escape. To overcome this, recent research proposes combining ADCs with distinct antibodies but shared payloads. Adding a TROP2-directed ADC, such as datopotamab deruxtecan (Dato-DXd), allows payload delivery via a HER2-independent pathway. Preclinical data suggest that low-dose combinations of T-DXd and Dato-DXd can achieve antitumor efficacy comparable to or greater than maximal doses of either agent alone, potentially reducing toxicity and improving tolerability [70,71].

Furthermore, for the HR-positive/HER2-positive subgroup, the 2024 Phase III PATINA trial showed a significant PFS benefit by adding the CDK4/6 inhibitor, palbociclib, to maintenance therapy, improving progression-free survival and potentially overcoming resistance, further refining precision care [72].

Collectively, these advances indicate that HER2 biology in metastatic breast cancer is no longer adequately captured by a strictly binary classification of HER2-positive versus HER2-negative disease. Therapeutic responsiveness increasingly reflects a continuum of HER2 expression, shaped by intratumoural heterogeneity, dynamic modulation under treatment pressure, and the pharmacological properties of modern antibody–drug conjugates. This evolving perspective has prompted a reassessment of HER2-negative disease and underpins the emergence of new, clinically actionable HER2 expression subcategories.

Recent advancements have challenged the traditional binary classification of HER2 status (positive vs. negative). The therapeutically actionable category of HER2-low has emerged, defined as tumors with an IHC score of 1+ or an IHC 2+ score with a negative ISH result [66,73]. This re-classification was prompted by the remarkable efficacy of next-generation antibody–drug conjugates (ADCs) such as trastuzumab-deruxtecan (T-DXd) [74]. This ADC is an engineered molecule that combines a HER2-targeting antibody with a potent cytotoxic chemotherapy payload via a cleavable linker. This design allows the drug to not only kill the target cells but also to diffuse and kill adjacent tumor cells with low or no HER2 expression, a critical mechanism known as the “bystander effect” [74]. This effect is dictated by the ADC’s specific molecular architecture: T-DXd possesses a high drug-to-antibody ratio (DAR) of approximately 8:1, utilizing a cathepsin-cleavable peptide linker [75]. Upon lysosomal cleavage within the target cell, the released payload—a highly membrane-permeable topoisomerase I inhibitor (DXd)—diffuses into the surrounding tumor microenvironment. This mechanistic feature ensures the potent elimination of neighboring tumor cells, regardless of their individual HER2 expression levels [74,75].

More recently, attention has focused on an emerging HER2-ultralow phenotype. This category refers to tumors historically classified as HER2-zero but exhibiting faint, focal HER2 staining in a very limited proportion of tumor cells. Although this phenotype remains under active clinical investigation, early evidence suggests that even minimal HER2 expression may be sufficient to permit ADC binding, internalization, and payload delivery. These observations further reinforce the concept of HER2 expression as a biological and therapeutic continuum rather than a set of rigid categorical thresholds.

The landmark DESTINY trial demonstrated a significant progression-free survival (PFS) benefit of approximately 5 months (9.9 vs. 5.1 months) with T-DXd compared to standard chemotherapy in previously treated HER2-low MBC, firmly establishing ADCs as a new standard of care in this setting [54]. For pathologists, these developments created a new, critical requirement to meticulously distinguish between IHC 0 (“HER2-zero”) and IHC 1+ staining, a distinction that was previously clinically irrelevant, but now directly determines therapeutic eligibility [41]. This challenge is compounded by evidence indicating that HER2-low status is highly dynamic, with discordance rates approaching 50% between primary and metastatic sites, underscoring the necessity of reassessment at disease progression [41,45].

### 3.5. PIK3CA Mutations

The phosphatidylinositol 3-kinase (PI3K) pathway is a crucial intracellular signaling cascade that promotes cell growth, proliferation, and survival [76]. Activating mutations in the *PIK3CA* gene, which encodes the p110-alpha catalytic subunit of PI3K [76], are the most common genetic alteration in HR+/HER2− breast cancer, found in approximately 40% of advanced cases [3,39,76]. These mutations are key oncogenic drivers and are associated with a poorer prognosis [77], leading to resistance to endocrine therapy [13,78].

This biomarker is therapeutically actionable. The approval of the PI3K-alpha inhibitor alpelisib, based on the SOLAR-1 Phase III trial, provided the first targeted therapeutic option for this population [20]. The trial demonstrated that adding alpelisib to fulvestrant, a potent SERD, significantly improved progression-free survival [54]. More recently, in October 2024, the FDA approved the next-generation, highly selective PI3K-alpha inhibitor inavolisib [14]. Inavolisib was evaluated in the Phase III INAVO120 trial, where its combination with palbociclib and fulvestrant more than doubled progression-free survival (PFS), reaching 17.2 months versus 7.3 months in the placebo group, with an HR of 0.42. The study also demonstrated a significant overall survival (OS) benefit, with 34.0 months for the inavolisib arm versus 27.0 months in the control group [79].

Despite these clinical gains, these inhibitors are associated with significant dose-limiting toxicities. Hyperglycemia is the most prevalent adverse event, occurring in up to 85% of patients treated with inavolisib and alpelisib due to the fundamental role of PI3K in insulin signaling. In the INAVO120 trial, Grade 3/4 hyperglycemia affected 5.6% of participants, often requiring proactive management with metformin. Other common toxicities include stomatitis (51%), rash, and diarrhea, which can lead to dose interruptions in approximately 28% of cases [53].

Furthermore, the durability of response is often limited by the development of acquired resistance. Recent genomic analyses identified that 50% of resistance is driven by alterations within the pathway, such as *PTEN* loss and activating *AKT1* mutations. Notably, secondary ‘pocket’ mutations in *PIK3CA* (e.g., in the kinase or helical domains) can emerge, which alter the binding affinity of orthosteric inhibitors [80]. Emerging evidence also suggests that while mTORC1 overactivation suppresses autophagy, its induction serves as a critical non-genomic survival mechanism for cancer cells under PI3K inhibition, representing a key escape route [81].

From a pathology perspective, while the majority of *PIK3CA* mutations occur in well-defined hotspot regions (e.g., exons 9 and 20) [82], a significant fraction also occur in non-hotspot regions [39]. Importantly, studies, including the SOLAR-1 trial, showed that patients with both hotspot and non-hotspot mutations appear to benefit from PI3K inhibition [54], justifying comprehensive genomic profiling (NGS) to detect all alterations and maximize patient eligibility [83]. Furthermore, *PIK3CA* mutations are considered early, “truncal” events in tumorigenesis [39]. This is reflected in a high concordance rate of approximately 90% (overall discordance rate of 9.8%) between primary tumors and metastatic sites, as confirmed in a large 2023 meta-analysis [39]. This stability is a key practical distinction from acquired mutations (like *ESR1*) and means that testing the archival primary tumor block is a reliable option if a new biopsy of the metastasis is not feasible [39].

### 3.6. AKT1/PTEN Alterations

While *PIK3CA* mutations (discussed in 3.3) are the most common alteration in the PI3K signaling pathway, alterations in other key nodes, particularly AKT1 and PTEN, also drive endocrine resistance [78]. The tumor suppressor gene *PTEN*, when lost or mutated, leads to hyperactivation of the pathway downstream, favoring activating mutations in *AKT1* [22,78].

This biomarker group became clinically actionable in late 2023 with the FDA approval of the first-in-class AKT inhibitor, capivasertib [22,84]. Based on the Phase III CAPItello-291 trial, the approval applies to patients with HR-positive, HER2-negative MBC whose tumors are eligible based on the presence of one or more alterations in the *PIK3CA*/*AKT1*/*PTEN* pathway [22,84]. The trial showed that the therapeutic combination of capivasertib with fulvestrant significantly improved progression-free survival in patients who had progressed on endocrine therapy [22].

For a pathologist’s guide, this creates a critical distinction:

- PI3K-alpha inhibitors (alpelisib, inavolisib) target *PIK3CA*-mutant tumors.
- The AKT inhibitor (capivasertib) targets tumors with *PIK3CA*, *AKT1*, or *PTEN* alterations.

Therefore, patients with *AKT1* or *PTEN* alterations *only* are candidates for capivasertib, but not for the PI3K-alpha-specific inhibitors [78]. This underscores the necessity for comprehensive NGS panels that assess all three genes, as recommended by NCCN and ESMO guidelines, to fully exhaust targeted therapy options for the HR-positive population [29,78].

### 3.7. ESR1 Mutations

Mutations in the *ESR1* gene, which encodes the ER-alpha protein, are a well-established mechanism of acquired resistance to endocrine therapy, particularly aromatase inhibitors (AIs) [36,40], but also associated with reduced sensitivity to other endocrine therapies, including SERMs and SERDs. These mutations, with D538G and Y537S being the most frequently observed in metastatic, endocrine-resistant breast cancer are rarely found in primary, untreated tumors (<5%) [40] but are selected by therapeutic pressure, emerging in up to 40% of patients with HR+/HER2− MBC following progression on AI therapy [36,40].

*ESR1* mutations in the ligand-binding domain lead to a constitutively active (ligand-independent) estrogen receptor. This renders the cancer cells independent of estrogen and thus resistant to therapies that block estrogen production (i.e., aromatase inhibitors) [36].

The identification of an *ESR1* mutation is a critical predictive biomarker. It signals resistance to AIs [36] and, most importantly, predicts benefit from a new generation of oral selective estrogen receptor degraders (SERDs). The FDA approval of elacestrant in 2023, based on the EMERALD trial, was the first approval in this class [54]. This field has advanced rapidly, with the subsequent September 2025 FDA approval of imlunestrant based on the positive EMBER-3 trial [21,55]. Imlunestrant, an oral SERD, demonstrated superior PFS in patients with ESR1 mutations in the EMBER-3 trial (5.5 vs. 3.8 months, HR 0.62) and showed additional synergy when combined with abemaciclib [55]. Data from recent trials for other oral SERDs, such as camizestrant (SERENA-6) and vepdegestrant (VERITAC-2), have further confirmed the central role of *ESR1* as an actionable target [55].

For pathologists, the dynamic and *acquired* nature of these mutations dictates the testing method. Unlike the stable, “truncal” *PIK3CA* mutations, testing the archival primary tumor for *ESR1* is clinically inappropriate as it will not reflect the tumor’s current resistance mechanisms. This makes *ESR1* mutations an ideal target for liquid biopsy (ctDNA) detection at the time of disease progression [40]. ctDNA analysis has shown high sensitivity and high concordance (97%) with contemporaneous metastatic tissue biopsies, establishing it as the preferred, non-invasive standard for guiding these critical treatment decisions [36,40].

### 3.8. BRCA1/2 and Other HRR Genes

The *BRCA1* and *BRCA2* genes are tumor suppressor genes that play a critical role in the homologous recombination repair (HRR) pathway for repairing double-strand DNA breaks [18]. Pathogenic mutations in these genes lead to a deficiency in this repair mechanism, a state known as homologous recombination deficiency (HRD) [18].

As a result, homologous recombination deficiency renders tumor cells sensitive to poly-(ADP-ribose) polymerase inhibitors (PARPi), through a mechanism known as “synthetic lethality” [85,86]. PARP inhibitors block the repair of single-strand DNA breaks; in HRD-impaired cells, these unresolved single-strand breaks degrade into double-strand breaks. The cell, unable to repair these via HRR, accumulates overwhelming DNA damage, leading to cell death [85,86].

This molecular signature is a standard-of-care predictive biomarker in HER2-negative MBC [29]. Two PARP inhibitors, olaparib (OlympiAD trial) and talazoparib (EMBRACA trial), are FDA-approved for the treatment of patients with HER2-negative MBC who have pathogenic or suspected pathogenic germline *BRCA1/2* mutations (gBRCAm) [18,29]. Therefore, germline testing for *BRCA1/2* is recommended for all patients with HER2-negative MBC to determine eligibility [18,29].

While the primary approvals are for germline mutations, the clinical utility of PARP inhibitors is expanding. Guidelines such as ESMO and ASCO also recommend considering testing for somatic (tumor) *BRCA1/2* mutations, as these patients also benefit (ESCAT II-B) [18,29]. Furthermore, clinical data have shown high response rates in patients with germline mutations in other HRR genes, most notably PALB2 [18,29]. Based on these data, NCCN Guidelines (Version 2025) state that PARP inhibitors “may be considered” for patients with germline *PALB2* mutations, solidifying its role as an actionable biomarker [87]. Broader HRD testing, which includes other pathway genes like *ATM* and *CHEK2*, remains an area of active investigation to identify other patient populations who may benefit from this class of drugs [18].

### 3.9. PD-L1 Expression

Programmed Death-Ligand 1 (PD-L1) is a protein expressed on cancer cells and other immune cells that helps tumors evade the body’s immune system [88]. In triple-negative breast cancer (TNBC), an aggressive subtype often characterized by a high number of tumor-infiltrating lymphocytes (TILs), PD-L1 expression serves as the key predictive biomarker for eligibility for immune checkpoint inhibitors (ICIs) [59].

The FDA approval of pembrolizumab in combination with chemotherapy for metastatic TNBC was based on the Phase III KEYNOTE-355 trial [59]. This trial demonstrated a significant improvement in progression-free survival (PFS) and overall survival (OS) in patients whose tumors expressed PD-L1 [59].

From a pathologist’s perspective, the scoring methodology is critical. The FDA-approved companion diagnostic for pembrolizumab is the PD-L1 IHC 22C3 pharmDx assay [59]. Eligibility is determined not by positivity on tumor cells alone, but by the Combined Positive Score (CPS) [59]. The CPS is calculated as the number of all PD-L1-positive cells (including tumor cells, lymphocytes, and macrophages) divided by the total number of viable tumor cells, multiplied by 100 [59].

A CPS ≥ 10 is the established cutoff for identifying patients who derive significant benefit from this therapy [59]. Approximately 40% of metastatic TNBCs meet this CPS ≥ 10 threshold [59].

As a biomarker, PD-L1 is known to be heterogeneous, with expression varying between different areas of the tumor and between different antibody clones (e.g., SP142, SP263, 22C3) [89,90]. Furthermore, its expression is dynamic and can change under therapeutic pressure. Meta-analyses have reported an overall discordance rate in PD-L1 expression between primary and metastatic sites of approximately 28% to 41% [91]. This instability strongly supports the guideline-mandated practice of testing a metastatic lesion whenever feasible, as the primary tumor’s status may not accurately reflect the current immune microenvironment of the advanced disease [90].

#### Tumor-Infiltrating Lymphocytes

In parallel to PD-L1, assessment of the tumor microenvironment provides an additional, pathology-accessible layer of biological context. Tumor-infiltrating lymphocytes (TILs) reflect the presence of an endogenous anti-tumor immune response within the tumor stroma and can be viewed as a pragmatic surrogate of “immune engagement” in breast cancer. In immunogenic subtypes such as TNBC—and in HER2-positive disease, where immune activity also contributes to therapeutic responsiveness—TIL assessment can complement Other emerging targets arePD-L1 by capturing a broader immune landscape rather than a single inhibitory ligand.

A practical advantage of TIL evaluation is that it can be performed on routine histopathology sections as part of standard pathological review, supporting its use as a cost-effective adjunct to biomarker-driven decision-making. In the metastatic setting, where biomarker heterogeneity and temporal evolution are central challenges, parallel consideration of immune contexture (such as TILs) aligns conceptually with the wider principle of re-testing and re-characterization at progression, similarly to the approach recommended for dynamic biomarkers such as PD-L1 [89,90].

As with HER2-low and PD-L1 scoring, the clinical utility of microenvironment-based metrics depends on standardized interpretation and robust quality assurance. Therefore, incorporating TIL assessment into routine reporting—when clinically relevant—should be supported by clear institutional workflows, harmonized scoring approaches, and integration into multidisciplinary discussions to ensure consistent interpretation alongside established predictive biomarkers [92].

### 3.10. EGFR

EGFR is overexpressed in 15–45% of breast cancers and is an independent predictor of poor prognosis (Overall Survival). It is associated with larger tumors, lower hormone receptor levels, and resistance to systemic therapy [93]. While standard inhibitors have shown limited success, novel drug delivery methods like peptide-tagged cubosome nanocarriers are being developed. These 157 nm cubosomes deliver paclitaxel selectively to EGFR-overexpressing cells, showing 75% selective uptake and significantly suppressed tumor growth in vivo [94].

### 3.11. Tumor-Agnostic Biomarkers (MSI, TMB, and NTRK Fusions)

Beyond breast cancer-specific biomarkers, several “tumor-agnostic” alterations are now part of routine practice for metastatic disease, as their presence predicts response to specific drugs regardless of the tumor’s site of origin [29].

- Tumor Mutational Burden (TMB): TMB is a genomic biomarker that measures the total number of somatic mutations per megabase (mut/Mb) [17]. It serves as a proxy for neoantigen load, which may stimulate an immune response [17]. While breast cancer is generally a TMB-intermediate tumor, the FDA has granted tumor-agnostic approval for pembrolizumab in any solid tumor identified as TMB-High (TMB-H), defined as ≥10 mut/Mb [18]. Though rare, this status can identify select MBC patients who may benefit from an ICI [18].
- Microsatellite Instability (MSI): MSI, a state of deficient DNA mismatch repair (dMMR), is another established tumor-agnostic biomarker for ICI response [18]. Pathologically, dMMR is identified by the loss of expression of one or more mismatch repair proteins (MLH1, MSH2, MSH6, PMS2) by IHC [18]. This status is extremely rare in breast cancer (occurring in <2% of cases) but is highly predictive of response to ICIs like pembrolizumab and dostarlimab-gxly. Although microsatellite instability and mismatch repair deficiency are rare in breast cancer, their clinical relevance in the metastatic setting is disproportionate to their prevalence. MSI/dMMR tumors exhibit a high mutational burden and continuous neoantigen generation, rendering them particularly susceptible to immune checkpoint blockade. The tissue-agnostic approvals of pembrolizumab and dostarlimab have therefore positioned MSI/dMMR status as a definitive predictive biomarker for immunotherapy across solid tumors, including breast cancer, irrespective of histological subtype. Importantly, contemporary clinical guidelines no longer restrict MSI/dMMR testing to traditionally enriched tumor types such as colorectal or endometrial cancer. Instead, universal screening for MSI/dMMR is increasingly endorsed in advanced solid tumors to avoid missing rare but highly actionable cases. From a pathology perspective, this reinforces the value of incorporating MSI/dMMR assessment within comprehensive genomic profiling strategies, particularly when next-generation sequencing is already being performed for metastatic disease. In the context of metastatic breast cancer, MSI/dMMR exemplifies a critical paradigm shift: biomarker testing is guided not by frequency but by actionability. Identification of this alteration, even in a small subset of patients, can profoundly alter therapeutic strategy by enabling access to immune checkpoint inhibitors with the potential for durable clinical benefit [18].
- NTRK Fusions: A third critical, though exceptionally rare, tumor-agnostic biomarker is the presence of gene fusions involving NTRK1, NTRK2, or NTRK3 [18]. These fusions produce constitutively active TRK fusion proteins that are oncogenic drivers. Their detection is highly actionable, as they predict profound and durable responses to FDA-approved TRK inhibitors like larotrectinib and entrectinib [18].

For pathologists, comprehensive NGS panels performed on metastatic tissue are recommended by both ASCO and ESMO to ensure these rare but highly actionable tumor-agnostic alterations are not missed [18,95].

## 4. Liquid Biopsy and Circulating Tumor DNA (ctDNA)

### 4.1. Techniques and Platforms

Liquid biopsy is a non-invasive diagnostic technique that involves analyzing biological fluids, most commonly blood, to detect cancer-related biomarkers [96]. The two primary analytes for pathologists are Circulating Tumor Cells (CTCs) and circulating tumor DNA (ctDNA) [40]. While CTC enumeration, using systems like the FDA-approved CellSearch^®^ platform, has established prognostic value in MBC, ctDNA analysis has become the primary tool for genomic profiling [97].

ctDNA consists of small, cell-free DNA (cfDNA) fragments released by apoptotic and necrotic tumor cells into the bloodstream [96]. This technology offers a significant advantage over traditional tissue biopsy as it is minimally invasive and, critically, provides a real-time snapshot of the tumor’s *entire* genomic profile. It is capable of capturing spatial heterogeneity from all metastatic sites simultaneously, which is a major limitation of single-lesion tissue biopsies [28,34].

Various platforms are used for ctDNA analysis, each with different strengths. Highly sensitive, PCR-based methods like digital droplet PCR (ddPCR) are particularly effective for monitoring a *specific*, known, low-frequency mutation (e.g., tracking an *ESR1* mutation) [36]. In contrast, broader targeted next-generation sequencing (NGS) panels are the standard for comprehensive genomic profiling. These panels allow for the simultaneous detection of a wide array of actionable mutations (e.g., *PIK3CA*, *ESR1*, *AKT1*, *BRCA*) from a single blood draw, as recommended by both NCCN and ESMO guidelines [83,87]. More advanced techniques, such as shallow whole-genome sequencing (sWGS), can also be used to assess copy number aberrations (CNAs) and overall tumor fraction in the blood [98].

### 4.2. Clinical Integration: Monitoring, Resistance, and Detection of Mutations

The clinical integration of ctDNA analysis represents a significant leap forward, moving breast cancer management from a static, one-time diagnosis to a dynamic, continuous process [28,34]. Its applications fall into two main categories: monitoring disease burden and detecting specific genomic alterations.

- Disease and Response Monitoring: A core application of ctDNA is its use for serially monitoring tumor burden. Studies have consistently shown that changes in ctDNA levels closely reflect changes in tumor burden and correlate with outcomes [34]. Crucially, rising ctDNA levels can serve as an early warning sign of therapeutic resistance, often predicting disease progression with a median lead time of 8.9 months (and up to 2 years) before it becomes apparent on standard imaging [34,99]. This provides a critical window of opportunity to intervene before disease progression.
- Detection of Actionable Mutations: This dynamic monitoring capability is most valuable for detecting the emergence of acquired resistance mutations that arise under the selective pressure of systemic therapies. The archetypal example is the ESR1 mutation. As mentioned previously, these mutations are rare in primary tumors (<5%) but are selected by endocrine therapy (particularly aromatase inhibitors) and are present in up to 40% of patients with HR-positive MBC progressing on treatment [36,40].

For a pathologist, testing for *ESR1* at progression is essential, and ctDNA is the preferred method, as the archival primary tumor tissue is not informative [100]. Studies show ctDNA has high sensitivity and concordance (97%) with contemporaneous metastatic biopsies for *ESR1* detection [36]. Identifying an *ESR1* mutation is a predictive biomarker that signals resistance to AIs and, most importantly, guides the clinical decision to switch to a new class of FDA-approved oral selective estrogen receptor degraders (SERDs) [54,55].

ctDNA is also used to identify mutations arising early during tumorigenesis, such as the ones found in *PIK3CA*. In contrast to *ESR1*, *PIK3CA* mutations are retained as truncal alterations across tumor clones and are therefore highly stable between the primary tumor and metastasis, with a concordance rate of approximately 90% [39]. Therefore, while ctDNA is an excellent tool, if a metastatic tissue biopsy is not feasible, the primary tumor block remains a valid alternative for *PIK3CA* testing [39].

This proactive approach transforms clinical practice from a reactive to a predictive model, where treatment adjustments can be made in anticipation of resistance, potentially improving clinical outcomes by circumventing ineffective therapies [35]. The shift from a single, static biopsy to a continuous, dynamic view of the tumor’s evolving genome is one of the most profound developments in precision oncology [28].

### 4.3. Limitations and Future Prospects

Despite its promise, ctDNA testing has several key limitations. The primary challenge is sensitivity: ctDNA yield can be low or undetectable in patients with a low tumor burden, certain indolent histologies, or metastases confined to sites like bone [96]. Furthermore, a lack of standardization across analytical platforms and assays hinders widespread clinical adoption [101]. For pathologists, a critical interpretation challenge is distinguishing true tumor-derived mutations from Clonal Hematopoiesis of Indeterminate Potential (CHIP), where mutations in background blood cells can be a source of false-positive results [101].

However, future prospects are rapidly evolving. The most immediate application is in early-stage breast cancer for the detection of minimal residual disease (MRD). Studies have shown that ctDNA detection after curative-intent surgery is a powerful prognostic tool, predicting metastatic relapse with a median lead time of 8.9 months before it becomes apparent on standard imaging [99]. Clinical trials are now underway to determine if escalating therapy based on MRD detection can improve outcomes [102]. Other future applications include population-level screening and analyzing epigenetic signatures, such as ctDNA methylation patterns, to dynamically track tumor evolution without prior genomic data [28,101].

## 5. Biomarker Testing Guidelines and Recommendations

### 5.1. Current Guidelines from ASCO, ESMO, NCCN, CAP

Major international guidelines from organizations such as the American Society of Clinical Oncology (ASCO) [18], the European Society for Medical Oncology (ESMO) [29], the National Comprehensive Cancer Network (NCCN) [63], and the College of American Pathologists (CAP) provide the framework for biomarker testing in metastatic breast cancer (MBC) [65].

A foundational consensus exists across all major guidelines recommending that, at the time of first recurrence, a metastatic lesion should be biopsied whenever feasible [18,29]. The primary purpose of this re-biopsy is to confirm the metastatic diagnosis and, critically, to re-evaluate the status of hormone receptors (ER, PR) and HER2, given the high rates of clinically significant discordance [29,38]. The joint ASCO/CAP guidelines provide the standardized scoring criteria for ER, PR, and the newly critical HER2-low category, forming the basis for pathologic assessment [66].

Beyond these foundational IHC markers, there is a clear consensus to perform comprehensive biomarker testing for all patients with MBC, stratified by subtype [29,95].

- For HR+/HER2− MBC: Guidelines universally recommend genomic profiling of the tumor (from tissue or ctDNA) at progression to identify actionable mutations, including PIK3CA, ESR1, and alterations in the PIK3CA/AKT1/PTEN pathway [29,103].
- For HER2-Negative MBC: Guidelines recommend germline *BRCA1/2* testing for all patients with HER2-negative MBC (both HR+ and TNBC) to determine eligibility for PARP inhibitors [29,103].
- For Triple-Negative MBC (TNBC): Testing for PD-L1 expression (using the 22C3 antibody and CPS scoring) is mandated to select patients for first-line immunotherapy with pembrolizumab [29].
- For All Subtypes: All guidelines recommend comprehensive genomic profiling to also assess for rare, tumor-agnostic biomarkers such as dMMR/MSI-H and NTRK fusions, which make patients eligible for specific targeted therapies [29,95].

To keep pace with this rapid evolution, guideline bodies have adopted new formats. The NCCN Biomarkers Compendium^®^ provides a continuously updated, evidence-based resource [63,103]. Similarly, ESMO now publishes a “Living Guideline” for metastatic breast cancer, which is updated in near-real-time as new trial data become available [29,104], and uses the ESMO Scale for Clinical Actionability of molecular Targets (ESCAT) to rank biomarkers [105].

### 5.2. Concordance and Variations Across Institutions

While there is strong alignment among major international guidelines (ASCO, ESMO, NCCN) on *which* biomarkers to test [18,29,104], significant institutional variations exist in the *implementation, interpretation, and application* of these recommendations [106].

The most prominent variation is in the routine performance of metastatic site re-biopsy. Although all major guidelines strongly recommend a biopsy of a metastatic lesion to re-evaluate ER, PR, and HER2 status, the logistical execution of this recommendation varies widely [29,63]. Institutional practices are often influenced by differences in available resources (e.g., access to interventional radiology), reimbursement policies, and provider consensus on the morbidity of the procedure versus the benefit of new information.

Furthermore, significant inter-observer and inter-institutional variability exists in the interpretation of the newer, more complex biomarkers:

- HER2-Low Scoring: The re-classification of “HER2-low” (IHC 1+ or 2+/ISH-negative) has introduced a major challenge for pathologic concordance [41,45]. Distinguishing a true IHC 0 (“HER2-zero”) from a faint or focal IHC 1+ is notoriously subjective and has shown high discordance rates in ring studies. This variability directly impacts patient eligibility for transformative ADC therapies [41].
- PD-L1 Scoring: PD-L1 testing in TNBC is another source of significant variation. Guidelines require the 22C3 clone and Combined Positive Score (CPS) for pembrolizumab, but different clones (e.g., SP142, SP263) and scoring algorithms exist for other indications, leading to potential confusion and a need for rigorous laboratory validation [91,107].
- ctDNA Platforms: As guidelines increasingly endorse ctDNA-based testing for acquired mutations like *ESR1*, institutions must choose between various platforms (e.g., broad NGS panels vs. specific ddPCR assays) [83]. This lack of standardization in assay selection, processing, and bioinformatic pipelines can lead to different results from the same blood sample [101].

Therefore, the successful implementation of modern biomarker guidelines requires more than just guideline awareness; it demands the establishment of new institutional workflows, robust internal and external quality assurance (QA) measures, and continuous educational initiatives for pathologists and oncologists to ensure concordant, high-quality results [95].

### 5.3. Challenges in Implementation

The translation of biomarker testing guidelines into routine clinical practice is challenging [106]. Key obstacles include economic and structural barriers, such as inconsistent patient access and insurance reimbursement for advanced genomic tests like multi-gene NGS panels [108,109].

From a health-systems perspective, these economic constraints are particularly relevant because the clinical utility of NGS is closely linked to how it is deployed within diagnostic pathways. When implemented early in metastatic disease, comprehensive genomic profiling can consolidate multiple biomarker assessments into a single workflow, supporting timely identification of actionable alterations and reducing delays associated with sequential testing strategies. This is aligned with the increasing emphasis by guideline bodies on broad profiling in advanced cancer to avoid missing rare but clinically meaningful targets [95].

In addition to clinical benefit, the economic rationale for NGS is increasingly framed around efficiency and resource allocation. The metastatic setting is characterized by complex treatment sequencing, and prolonged diagnostic pathways may delay access to effective targeted therapies, potentially increasing downstream costs associated with ineffective treatment exposure. Moreover, the ability of NGS-based approaches to identify tumor-agnostic biomarkers (such as MSI/dMMR and gene fusion) reinforces the principle that low-frequency findings can still have high clinical and economic impact when they enable access to effective targeted therapies [95].

Finally, the integration of ctDNA assays can further influence economic viability by reducing reliance on repeated invasive biopsies and supporting longitudinal monitoring of resistance evolution. However, implementation nowadays requires careful attention to assay choice, turnaround time (TAT), laboratory infrastructure, reimbursement frameworks, and other important factors such as harmonized interpretation standards. Importantly, all of these factors can drive variability across institutions [101].

The logistical difficulty of obtaining high-quality metastatic tissue remains a primary hurdle. The guideline recommendation to re-biopsy “whenever feasible” [29] is often challenging in practice, especially for patients with bone-only or difficult-to-access visceral metastases. Furthermore, technical issues, such as the decalcification of bone biopsies, can technically compromise the quality and reliability of IHC and molecular testing, underscoring the value of liquid biopsy (ctDNA) as an alternative [29].

Moreover, the multi-week turnaround time for some complex genomic assays can be a significant barrier [110]. This delay may not align with the time-sensitive nature of clinical decisions in a rapidly progressing disease setting, such as impending visceral crisis [29].

Finally, implementation is challenged by the need for continuous pathologist and oncologist education [92]. The rapid introduction of new, subjective scoring criteria (e.g., distinguishing HER2-zero from HER2-low) and complex companion diagnostics (e.g., PD-L1 CPS scoring) requires robust, institution-wide training and rigorous quality assurance (QA) protocols to ensure concordant, high-quality results [38,111].

## 6. Challenges and Limitations

### 6.1. Tumor Heterogeneity and Evolution

A central limitation of biomarker-guided therapy in metastatic breast cancer is that most diagnostic assays are inherently static whereas tumor biology is dynamic and spatially heterogeneous. The fundamental challenge is the presence of intratumour heterogeneity (ITH) and therapy-guided clonal evolution, which means that a single tissue biopsy, whether from the primary tumor or a metastatic lesion, provides only a partial and time-restricted representation of the disease biology at one location and one moment in time [26,101,112]. This limitation becomes clinically critical as tumors evolve under Darwinian selective pressure, leading to the emergence of new subclones with novel resistance mechanisms [26,28].

This continuous process of clonal evolution means that the biology and molecular profile of a progressing metastasis may no longer match the archival primary tumor from which it was derived [113]. Therefore, a static biomarker assessment performed at diagnosis is often insufficient to guide subsequent lines of therapy in the metastatic setting [28]. This core challenge necessitates a shift toward real-time, longitudinal and adaptive monitoring to map the tumor’s evolutionary trajectory, which is precisely the promise of liquid biopsy technologies [28,34]. In fact, liquid biopsy technologies, particularly the analysis of circulating tumor DNA (ctDNA), offer a complementary approach by enabling real-time, non-invasive tracking of clonal dynamics across all metastatic sites simultaneously [26,28].

### 6.2. Discordance Between Primary and Metastatic Sites

As previously discussed, relying on the biomarker status of the primary tumor can lead to a misdiagnosis of the current disease state and the selection of an ineffective therapy [37,114]. This challenge of temporal and spatial discordance is a direct consequence of tumor heterogeneity and clonal evolution under therapeutic pressure [38]. The magnitude of this challenge is significant, as documented in large-scale meta-analyses.

Pooled data show discordance rates between primary and metastatic sites are approximately 19–20% for ER [38,64,65], 33–34% for PR (the most unstable classic marker), and 8–15% for HER2-positive status [38,64]. This instability is even more pronounced for dynamic or subjective biomarkers, with discordance for PD-L1 expression reported between 28 and 41% [42,91] and the critical HER2-low classification approaching 50% [41,45].

This challenge is further divided into two distinct pathological scenarios:

1. Concordant (but stable) mutations, such as *PIK3CA*, which are “truncal” events and show high concordance (~90%) [39].
2. Acquired (dynamic) mutations, such as *ESR1*, which are absent at diagnosis but emerge in up to 40% of patients at progression [115].

This complex landscape of stable, unstable, and acquired biomarkers underscores the critical need for re-biopsy of metastatic lesions, as recommended by all major guidelines [29], or the use of liquid biopsy to obtain the most current and relevant molecular information [83].

### 6.3. Analytical and Preanalytical Issues

The reliability of all biomarker testing is highly dependent on standardized and validated assays, beginning with preanalytical sample handling [111]. For tissue biopsies, preanalytical issues such as prolonged cold ischemic time, improper tissue fixation, or variations in fixation duration can degrade antigens, leading to inaccurate false-negative results for ER, PR, and HER2 [65]. This is a particularly critical challenge for bone metastases, a common site of disease, as the decalcification process required for analysis can severely compromise or destroy both IHC and ISH signals [29].

For ctDNA analysis, preanalytical variables include the use of specialized blood collection tubes to prevent cfDNA degradation and leukocyte lysis [101]. Analytically, the low fraction of tumor DNA in some samples (e.g., bone-only disease) can fall below the assay’s limit of detection, posing a significant challenge and leading to false-negative results [101]. Furthermore, interpreting ctDNA results requires distinguishing true somatic mutations from Clonal Hematopoiesis of Indeterminate Potential (CHIP), where mutations arise from blood cells, not the tumor, creating a source of false-positive findings [101].

The need for rigorous quality assurance (QA) and the use of validated companion diagnostics (CDx) is paramount. This is exemplified by the subjective challenges in HER2-low scoring, where high inter-observer variability in distinguishing IHC 0 from IHC 1+ directly impacts treatment eligibility [41]. Similarly, different IHC clones for PD-L1 (e.g., 22C3 vs. SP142) are not interchangeable and are tied to specific drugs and scoring systems (e.g., CPS), creating a complex analytical landscape that requires meticulous validation by the pathology lab [91,107].

Beyond tumor-intrinsic biomarkers, patient-specific pharmacogenetic factors represent an increasingly important dimension of precision oncology. Deficiency of dihydropyrimidine dehydrogenase (DPD), encoded by the DPYD gene, is a well-established cause of severe and potentially life-threatening toxicity in patients treated with fluoropyrimidines, including 5-fluorouracil and capecitabine. Even standard doses can lead to profound hematologic, gastrointestinal, and neurological adverse events in individuals with partial or complete DPD deficiency [116].

Prospective genotyping of DPYD polymorphisms (notably alleles *2A, *13, and c.2846A > T) is now a clinical imperative. Both CPIC and ESMO guidelines mandate testing prior to the initiation of chemotherapy to facilitate preemptive dose adjustments or the selection of alternative treatment regimens [117]. From an implementation standpoint, DPYD testing illustrates how precision medicine extends beyond tumor genomics to encompass patient-specific determinants of treatment tolerability. Integrating pharmacogenetics into oncological workflows improves patient safety and optimizes therapeutic delivery, particularly in the metastatic setting where cumulative toxicity and treatment sequencing are key considerations.

### 6.4. Integration of Multi-Omics Testing in Routine Practice

In the era of precision oncology, multi-omics integration should not be interpreted as the simultaneous deployment of all possible omics technologies, but rather as the structured synthesis of genomics (from tissue and circulating tumor DNA), key immunohistochemical biomarkers, and clinical information in a clinically actionable framework. The era of precision oncology is moving beyond single-analyte tests toward multi-omics profiling, which integrates data from genomics (DNA), transcriptomics (RNA), proteomics (protein), and other fields [17,118]. This shift is driven by the recognition that genomics alone is often insufficient to capture the full complexity of a tumor’s biology, which is also dictated by gene expression, protein function, and the tumor microenvironment [26].

The challenge lies in synthesizing these complex, high-dimensional datasets into a single, actionable clinical report that can be readily interpreted by a clinician [17,25]. In practice, this challenge relates less to data generation than to clinical prioritization and interpretation, particularly as molecular testing increasingly identifies alterations of uncertain or context-dependent significance. As this technology moves from research into routine practice, it requires a new infrastructure. This includes sophisticated bioinformatic tools and artificial intelligence (AI) algorithms to filter, integrate, and find patterns within the data that are not visible in any single dataset [119,120]. Furthermore, it necessitates the widespread implementation of expert molecular tumor boards (MTBs), which provide a comprehensive, multi-disciplinary review of each patient’s case to translate this complex data into a clear treatment recommendation [29].

## 7. Emerging and Investigational Biomarkers

### 7.1. HER3 and FGFR Alterations

HER3 and FGFR alterations are highlighted here not as an exhaustive overview of emerging targets, but as representative examples of biologically validated resistance-associated pathways that are already being translated into late-phase clinical trials and antibody–drug conjugate strategies in metastatic breast cancer. Beyond the established biomarkers discussed in Section 3, a new generation of molecular targets is in late-stage investigation, offering the potential to overcome therapeutic resistance, particularly in the HR-positive setting.

- HER3 (ERBB3): While not typically a primary oncogenic driver itself, HER3 is frequently overexpressed in endocrine-resistant HR-positive breast cancer and has emerged as a highly promising therapeutic target [7]. The focus is on HER3-directed antibody–drug conjugates (ADCs). Patritumab deruxtecan (HER3-DXd), in particular, has shown significant activity in heavily pre-treated patients with HR+/HER2− MBC [121]. Recent pivotal data from the HERTHENA-Breast01 and ICARUS-BREAST01 trials have validated its efficacy profile in this setting, while recent 2025 data from the TUXEDO-3 trial also showed intracranial responses in patients with active brain metastases, addressing a critical unmet need [122].
- FGFR Alterations: Alterations in the fibroblast growth factor receptor (FGFR) pathway, most commonly FGFR1 amplification (comprising 5–10% of HR+ breast cancers), are a well-established mechanism of *de novo* and acquired resistance to endocrine therapy [119,120]. The identification of these alterations by NGS (from tissue or ctDNA) is being actively investigated in numerous clinical trials. These trials are exploring the addition of selective FGFR inhibitors to standard endocrine-based regimens (such as CDK4/6 inhibitors) to re-sensitize tumors or prevent the onset of resistance [123].

Other emerging targets are under active investigation but remain earlier in translational development.

### 7.2. Spatial Transcriptomics, Proteomics, and AI-Based Predictors

The next frontier of biomarker discovery is moving beyond single-gene genomics toward multi-omics profiling, integrating data from genomics (DNA), transcriptomics (RNA), and proteomics (protein) [17,118]. This shift is driven by the recognition that genomics alone is often insufficient to capture the full complexity of a tumor’s biology, which is also dictated by gene expression and protein function within the tumor microenvironment [26].

Cutting-edge technologies are now enabling this integration with unprecedented detail and spatial resolution. Spatial transcriptomics enables the high-resolution, location-specific mapping of gene expression, while spatial proteomics (e.g., multiplex immunofluorescence) allows for the in situ visualization of multiple proteins within preserved tissue architecture [89]. These tools reveal the intricate interactions between cancer cells and their surrounding immune and stromal cells, unlocking novel biomarkers and deciphering mechanisms of treatment resistance (like T-cell exclusion) that are invisible to traditional bulk sequencing methods [124].

The challenge lies in synthesizing these complex, high-dimensional datasets into a single, actionable clinical report [17,25]. This is where Artificial Intelligence (AI) is being harnessed. For pathologists, the most transformative application is the use of deep learning models to predict molecular data directly from standard histopathology (H&E) slides [125,126]. As highlighted at recent conferences like ESMO 2025, AI models can provide a fast, cost-effective method to predict genomic alterations and identify high-risk mutations from the H&E image alone [125].

As these technologies move from research to practice, they will require a new infrastructure, including sophisticated bioinformatic tools and expert molecular tumor boards (MTBs), to provide a comprehensive, multi-disciplinary review of each patient’s case, further advancing the goal of truly personalized oncology [29].

## 8. Conclusions and Future Directions

The diagnostic landscape in metastatic breast cancer has evolved from foundational markers to a complex ecosystem, requiring the precise identification of HER2-low status [24] and actionable genomic alterations such as PIK3CA/AKT1 [22], acquired ESR1 mutations [54], and germline BRCA1/2 [58] variants.

Crucially, biomarker discordance between primary and metastatic sites has emerged as a critical challenge in this new era. Relying solely on the molecular profile of the primary tumor can misrepresent the current disease state, potentially leading to suboptimal therapy selection. Continuous monitoring of these biomarker dynamics throughout disease progression is therefore essential to tailor treatments effectively. This shift elevates the pathologist from a morphological observer to a “molecular gatekeeper,” whose role is best executed within expert Molecular Tumor Boards [127] to synthesize these diverse data streams.

Future precision oncology will depend on the seamless integration of multi-omics profiling, leveraging ctDNA liquid biopsy [128] for real-time monitoring of resistance and spatial transcriptomics [129] to map the tumor microenvironment. Furthermore, AI-based predictive models [126] applied to standard H&E slides are emerging as powerful tools to screen for biomarkers and augment—not replace—pathologists, moving the field toward a truly adaptive and proactive therapeutic strategy.

## Figures and Tables

**Table 1:** Five-Year Relative Survival Rates for Distant-Stage (Metastatic) Breast Cancer by Molecular Subtype.

Subtype	5-Year Relative Survival Rate
HR+/HER2+	45–48%
HR−/HER2+	40–42%
HR+/HER2−	38–41%
Triple-Negative (HR−/HER2−)	14–16%

Source: SEER 21 Data, **latest consolidated releases** 2018–2022 diagnosis.

**Table 2:** Key Clinically Actionable Biomarkers in Metastatic Breast Cancer.

Biomarker/Gene	Associated Subtype	Utility/Rationale for Testing	Key Associated Therapies/Therapy Class	Primary Testing Method
**ER, PR**	HR+	Guides endocrine therapy (ET). Re-testing at metastasis is critical due to discordance (25.1% for ER, 33.3% for PR) [46,47].	Endocrine Therapy (e.g., AIs, SERDs) + CDK4/6 inhibitors [48,49,50].	IHC
**HER2 (Positive)**	HER2+ (IHC 3+ or IHC 2+/ISH+)	Predictive of response to anti-HER2 therapies. Re-testing at metastasis is critical due to discordance (32.8%) [46,47].	T-DXd + Pertuzumab (1L), THP (Trastuzumab/Pertuzumab/Taxane) [51,52]. Palbociclib (for HR+/HER2+) [50].	IHC (ISH if 2+)
**HER2-Low**	HER2-Negative (IHC 1+ or IHC 2+/ISH-neg)	Predictive of response to specific ADCs. Critical to re-test metastatic site due to high discordance (~50%) [46].	Trastuzumab deruxtecan (T-DXd) [24].	IHC (Requires meticulous distinction of 0 vs. 1+)
***PIK3CA* Mutations**	HR+/HER2− (~40% of cases)	Predictive of response to PI3K-alpha inhibitors. High concordance (~90%) allows testing of primary or metastatic tissue.	Alpelisib, Inavolisib (approved Oct 2024) [20,53].	NGS (Tissue or ctDNA)
***AKT1*/*PTEN* Alterations**	HR+/HER2−	Predictive of response to AKT inhibitors. Guideline-recommended test for HR+ progression.	Capivasertib (approved 2023) [22].	NGS (Tissue or ctDNA)
***ESR1* Mutations**	HR+/HER2−	Identifies acquired resistance to mainly AIs, but also to Hormonal Therapy. Emerges in ~40% of patients post-AI therapy.	Oral SERDs: Elacestrant, Imlunestrant (approved Sep 2025) [54,55].	ctDNA (Liquid Biopsy) at progression
**Germline *BRCA1*/*2* and *PALB2***	HER2-Negative (HR+ or TNBC)	Predictive of response to PARP inhibitors. *PALB2* now considered actionable by NCCN [56].	PARP Inhibitors: Olaparib, Talazoparib [57,58].	NGS (Germline test)
**PD-L1 Expression**	TNBC	Predictive of response to immunotherapy. Requires specific 22C3 clone and CPS ≥ 10.	Pembrolizumab (+ Chemotherapy) [59].	IHC (22C3 clone, CPS scoring)
**MSI-H/dMMR**	Tumor-Agnostic (Rare in breast)	Tumor-agnostic predictive biomarker for ICI response.	Pembrolizumab, Dostarlimab-gxly [60].	IHC (for dMMR), NGS (for MSI)
**TMB-High (TMB-H)**	Tumor-Agnostic	Tumor-agnostic predictive biomarker (cutoff ≥ 10 mut/Mb) for ICI response.	Pembrolizumab [60].	NGS (Tissue)
***NTRK* Fusions**	Tumor-Agnostic (Extremely rare)	Tumor-agnostic predictive biomarker for TRK inhibitors.	Larotrectinib, Entrectinib [61].	NGS, IHC (pan-TRK)

Legend: AI (Aromatase Inhibitor); ADC (Antibody–Drug Conjugate); ASCO (American Society of Clinical Oncology); CAP (College of American Pathologists); CPS (Combined Positive Score); ctDNA (Circulating Tumor DNA); dMMR (Mismatch Repair Deficient); ER (Estrogen Receptor); ESMO (European Society for Medical Oncology); HER2 (Human Epidermal Growth Factor Receptor 2); HR (Hormone Receptor); IHC (Immunohistochemistry); ISH (In Situ Hybridization); MBC (Metastatic Breast Cancer); MSI-H (Microsatellite Instability-High); NCCN (National Comprehensive Cancer Network); NGS (Next-Generation Sequencing); PARP (Poly polymerase); PD-L1 (Programmed Death-Ligand 1); PR (Progesterone Receptor); SERD (Selective Estrogen Receptor Degrader); TMB (Tumor Mutational Burden); TNBC (Triple-Negative Breast Cancer).

## Data Availability

No new data were created or analyzed in this study. Data sharing is not applicable.

## Abbreviations

The following abbreviations are used in this manuscript:

ADC	Antibody–Drug Conjugate
AI	Artificial Intelligence
AIs	Aromatase Inhibitors
ASCO	American Society of Clinical Oncology
BRCA	Breast Cancer Gene
CAP	College of American Pathologists
CDK4/6	Cyclin-Dependent Kinase 4/6
CDx	Companion Diagnostic
cfDNA	Cell-Free DNA
CHIP	Clonal Hematopoiesis of Indeterminate Potential
CMF	Cyclophosphamide, Methotrexate, and Fluorouracil
CNAs	Copy Number Aberrations
CPS	Combined Positive Score
CTC	Circulating Tumor Cell
ctDNA	Circulating Tumor DNA
ddPCR	Digital Droplet PCR
dMMR	Deficient Mismatch Repair
ER	Estrogen Receptor
ESCAT	ESMO Scale for Clinical Actionability of Molecular Targets
ESMO	European Society for Medical Oncology
FDA	U.S. Food and Drug Administration
FGFR	Fibroblast Growth Factor Receptor
gBRCAm	Germline *BRCA1/2* Mutation
H&E	Hematoxylin and Eosin
HER2	Human Epidermal Growth Factor Receptor 2
HER3	Human Epidermal Growth Factor Receptor 3
HER3-DXd	Patritumab Deruxtecan
HR	Hormone Receptor
HRD	Homologous Recombination Deficiency
HRR	Homologous Recombination Repair
ICI	Immune Checkpoint Inhibitor
IHC	Immunohistochemistry
ISH	In Situ Hybridization
ITH	Intratumor Heterogeneity
MBC	Metastatic Breast Cancer
MRD	Minimal Residual Disease
MSI	Microsatellite Instability
MSI-H	Microsatellite Instability-High
MTB	Molecular Tumor Board
NCCN	National Comprehensive Cancer Network
NGS	Next-Generation Sequencing
OS	Overall Survival
PARP	Poly (ADP-ribose) Polymerase
PARPi	PARP Inhibitors
PD-L1	Programmed Death-Ligand 1
PFS	Progression-Free Survival
PI3K	Phosphatidylinositol 3-Kinase
PR	Progesterone Receptor
QA	Quality Assurance
SEER	Surveillance, Epidemiology, and End Results
SERD	Selective Estrogen Receptor Degrader
SERM	Selective Estrogen Receptor Modulator
sWGS	Shallow Whole-Genome Sequencing
TAT	Turnaround Time
T-DXd	Trastuzumab Deruxtecan
THP	Trastuzumab, Pertuzumab, and Taxane
TILs	Tumor-Infiltrating Lymphocytes
TMB	Tumor Mutational Burden
TMB-H	Tumor Mutational Burden-High
TNBC	Triple-Negative Breast Cancer
